# Supplementary material for: Impact of MMP-2 and MMP-9 enzyme activity on wound healing, tumor growth and RACPP cleavage
Source: PLoS One. 2018 Sep 24;13(9):e0198464. doi: 10.1371/journal.pone.0198464 (PMC6152858; doi:10.1371/journal.pone.0198464)
Supplement: S1 Table — (PDF) [file pone.0198464.s001.pdf]

S1 Table. Oligonucleotides

Gene	GenBank-EMBL number	Primer 1 (forward)	Primer 2 (reverse)
GAPDH	NM_008084	AGGTCGGTGTGAACGGATTTG	TGTAGACCATGTAGTTGAGGTCA
ß-actin	NM_007393	GGCTGTATTCCCCTCCATCG	CCAGTTGGTAACAATGCCATGT
Mmp1	NM_032006.3	CCACTGGTGATTTTGCCCAGA	CCGGTCTCCGTTGTGAAACA
Mmp2	NM_008610.2	CCCTGGTGGCTGGAGGCTCT	AACGGGGTCCCACGTCCCAA
Mmp3	NM_010809.1	GACCTAGAAGGAGGCAGCAG	AGGACCGGAAGACCCTTCAT
Mmp7	NM_010810.4	GCTGCCACCCATGAATTTGG	GCCTGCAATGTCGTCCTTTG
Mmp8	NM_008611.4	TTCCGGTCTTCGAGGAATGC	GTGTGTGTCCACTTGGGACT
Mmp9	NM_013599.3	CAGACGTGGGTCGATTCCAA	AGTAGTTTTGGATCCAGTATGTGA
Mmp10	NM_019471.2	GCACGAAGAAGGATCGGTTT	GCTGAGCAGATTGGTAGGCT
Mmp11	NM_008606.2	TGTACTGAATGCCCGGAACC	CCTCGTGCACCTCAGTGAAA
Mmp12	NM_008605.3	TTGGGCTAGAAGCAACTGGG	ATCCTCACGCTTCATGTCCG
Mmp13	NM_008607.2	ACAGGCTCCGAGAAATGCAA	CCACATCAGGCACTCCACAT
Mmp14	NM_008608.3	CGCGCTCTAGGAATCCACAT	CCCTGGAGGTAGGTAGCCAT
Mmp15	NM_008609.3	CAGACATCCCCTATGACCGC	CCATGCTGTGTCTCCTCGTT
Mmp17	NM_011846.4	TGAAAACCCCTCGATGCTCC	GTCCGGACCCTCCAAGAAAG
Mmp19	NM_021412.2	GTCTGCTGGGATCATGGACTG	CGTCATCCATCTGACCGGAAA
Mmp21	NM_152944.1	CCGCAAGGAGAGGAACCAAT	CCGTAGCGAGTCCTGTTGTT
Mmp23	NM_011985.2	ACCATGTCGGTAACCCGAAG	TCCACATTCGAAAGGCAGCA
Mmp24	NM_010808.3	GGATCCACTCGCCATCTGAG	CCTTCCTGCACCCGGTTATT
Mmp25	NM_001033339.3	CCGGACTACCTGAGACTGGA	CGGGCTCAACTGGGATTTCT
Mmp27	NM_001030289.1	GGCTGTCCCACTCCAATGAT	GACCCTCCATAAGTGCCTGC
Mmp28	NM_080453.2	GGCAATGTTTCAGAGCCTCG	CGGGAACCCTTGAAGAGGAC

Table S2. Breeding results for production of MMP-2 and -9 double KO mice. * $p < 0.05$ Chi-square test. The number of pups reflects those surviving to weaning at 3 weeks of age.

Year	1	2	3	Total
Number of litters	21	19	14	54
Ave litter size	4.95	4.74	4.5	4.76
Total # pups	104	90	63	257
# DKO pups	38	30	21	89 *

Table S3. Kinetic analysis of enzyme optimized RACPP with K_{cat}/K_m for selected enzymes.

Sequence	*Selectivity*	*MMP-2*	*MMP-9*	*MMP-12*	*MMP-14*	*Elastase*	*uPA*	*Thrombin*	*Plasmin*	*Cathepsin-K*	*Chymase*
**PLGC(me)AG**	MMP 2/9/12/14	36429	13503	9167	17173	4001	1438	-	-	2640	-
**TLSLEH**	MMP2	11405	-	1401	1200	-	-	-	-	497	2272

Enzymes that show no detectable activity with the substrate were left blank.

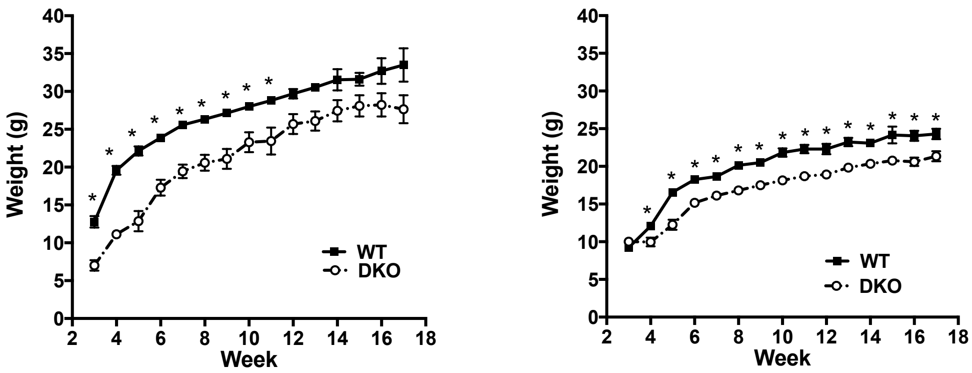

Figure S1. Growth is compromised in DKO mice. A. Average body weights over time of wild type (WT) and double KO (DKO) male mice. B. Average body weights over time of WT and DKO female mice. Data are means ± SEM analyzed by t tests using the Holm-Sidak correction for multiple comparisons. N = 7-14 mice per group. * p<0.001

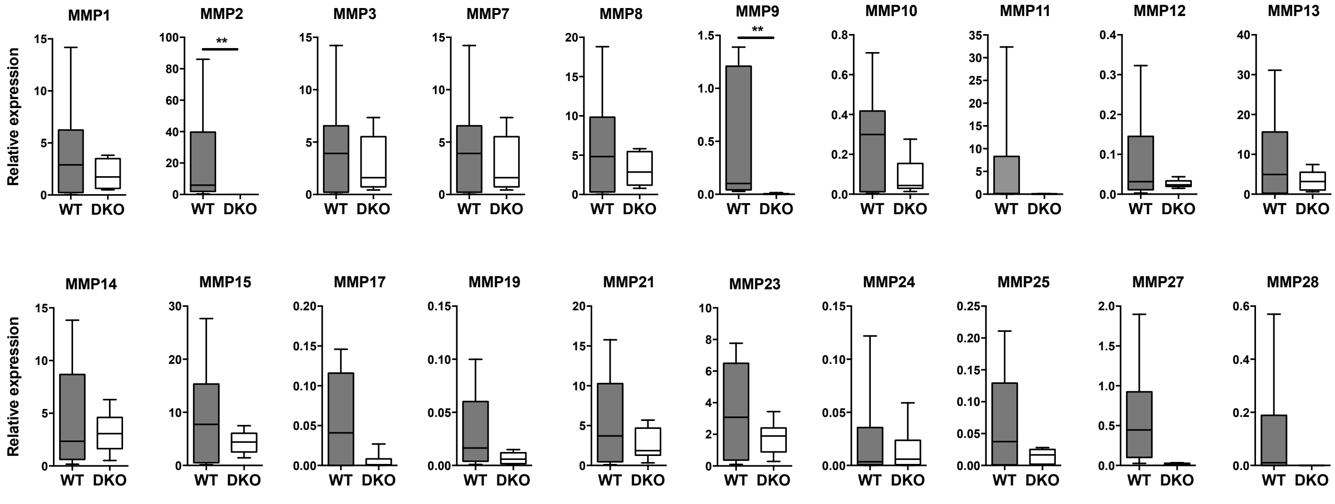

Figure S2. MMP-2 and -9 deletion was confirmed by RT-PCR. Semi-quantitative RT-PCR showing the gene expression of a panel of MMPs. MMP-2 and MMP-9 are the only MMPs with significant differences between the WT and DKO tumors. N = 6 tumors from PyVmT;WT or PyVmT;DKO mice. Data are box and whisker plots with min and max, ** p<0.01, Mann-Whitney test.

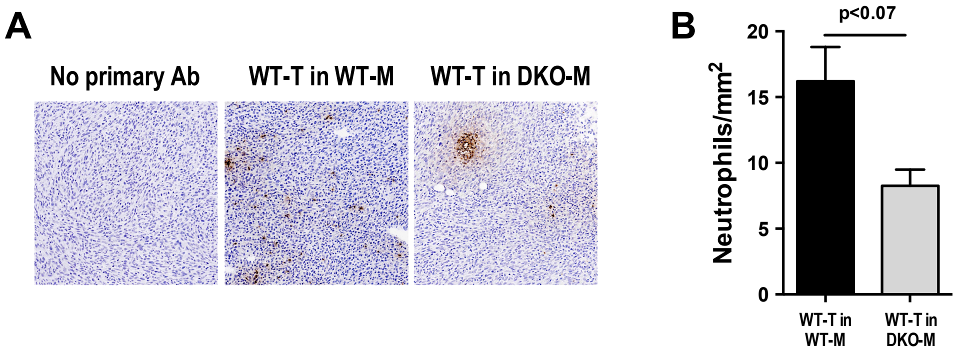

Figure S3. Neutrophil infiltration in WT and DKO tumors. A. Immunohistochemistry for neutrophil staining (NIMP-R14 antibody) of tumor sections with no primary antibody (control) as well as in WT-T in WT-M and DKO-M. B. The brown staining neutrophils from immunostained tumor sections were counted and represented per mm^2 of tumor area. N = 4-6 sections/group. Data are means ± SEM analyzed by Student's t test.
