# Supplementary material for: Impact of MMP-2 and MMP-9 enzyme activity on wound healing, tumor growth and RACPP cleavage
Source: PLoS One. 2018 Sep 24;13(9):e0198464. doi: 10.1371/journal.pone.0198464 (PMC6152858; doi:10.1371/journal.pone.0198464)
Supplement: S2 Table — * p < 0.05 Chi-square test. The number of pups reflects those surviving to weaning at 3 weeks of age. (PDF) [file pone.0198464.s002.pdf]

S2 Table. Breeding results for production of MMP-2 and -9 double KO mice.
The number of pups reflects those surviving to weaning at 3 weeks of age.
* $p < 0.05$ Chi-square test.

**Year**	**1**	**2**	**3**	**Total**
**Number of litters**	21	19	14	54
**Ave litter size**	4.95	4.74	4.5	4.76
**Total # pups**	104	90	63	257
**# DKO pups**	38	30	21	89 *
