# Supplementary material for: Impact of MMP-2 and MMP-9 enzyme activity on wound healing, tumor growth and RACPP cleavage
Source: PLoS One. 2018 Sep 24;13(9):e0198464. doi: 10.1371/journal.pone.0198464 (PMC6152858; doi:10.1371/journal.pone.0198464)
Supplement: S3 Table — (PDF) [file pone.0198464.s003.pdf]

S3 Table. Kinetic analysis of enzyme optimized RACPP with K_{cat}/K_m for selected enzymes.

Sequence	*Selectivity*	*MMP-2*	*MMP-9*	*MMP-12*	*MMP-14*	*Elastase*	*uPA*	*Thrombin*	*Plasmin*	*Cathepsin-K*	*Chymase*
**PLGC(me)AG**	MMP 2/9/12/14	36429	13503	9167	17173	4001	1438	-	-	2640	-
**TLSLEH**	MMP2	11405	-	1401	1200	-	-	-	-	497	2272

Enzymes that show no detectable activity with the substrate were left blank.
