# Supplementary material for: Impact of MMP-2 and MMP-9 enzyme activity on wound healing, tumor growth and RACPP cleavage
Source: PLoS One. 2018 Sep 24;13(9):e0198464. doi: 10.1371/journal.pone.0198464 (PMC6152858; doi:10.1371/journal.pone.0198464)
Supplement: S1 Fig — A. Average body weights of wild type (WT) and double KO (DKO) mice. B. Average body weights of WT and DKO female mice with individual body weights of DKO females on the right. Data are means ± SEM analyzed by t tests using the Holm-Sidak correction for multiple comparisons. N = 7–14 mice per group. * p<0.001. (PDF) [file pone.0198464.s004.pdf]

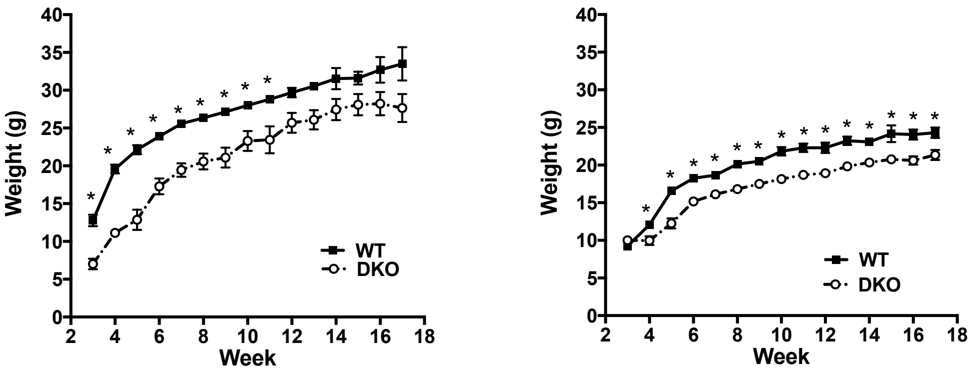

S1 Fig. Growth is compromised in DKO mice. A. Average body weights over time of wild type (WT) and double KO (DKO) male mice. B. Average body weights over time of WT and DKO female mice. Data are means ± SEM analyzed by t tests using the Holm-Sidak correction for multiple comparisons. N = 7-14 mice per group. * $p<0.001$
