# Supplementary material for: Impact of MMP-2 and MMP-9 enzyme activity on wound healing, tumor growth and RACPP cleavage
Source: PLoS One. 2018 Sep 24;13(9):e0198464. doi: 10.1371/journal.pone.0198464 (PMC6152858; doi:10.1371/journal.pone.0198464)
Supplement: S2 Fig — Semi-quantitative RT-PCR showing the gene expression of a panel of MMPs. MMP-2/-9 are the only MMPs with significant differences between the WT and DKO tumors. N = 6 tumors from PyVmT;WT or PyVmT;DKO mice. Data are box and whisker plots with min and max, ** p<0.01, Mann-Whitney test. (PDF) [file pone.0198464.s005.pdf]

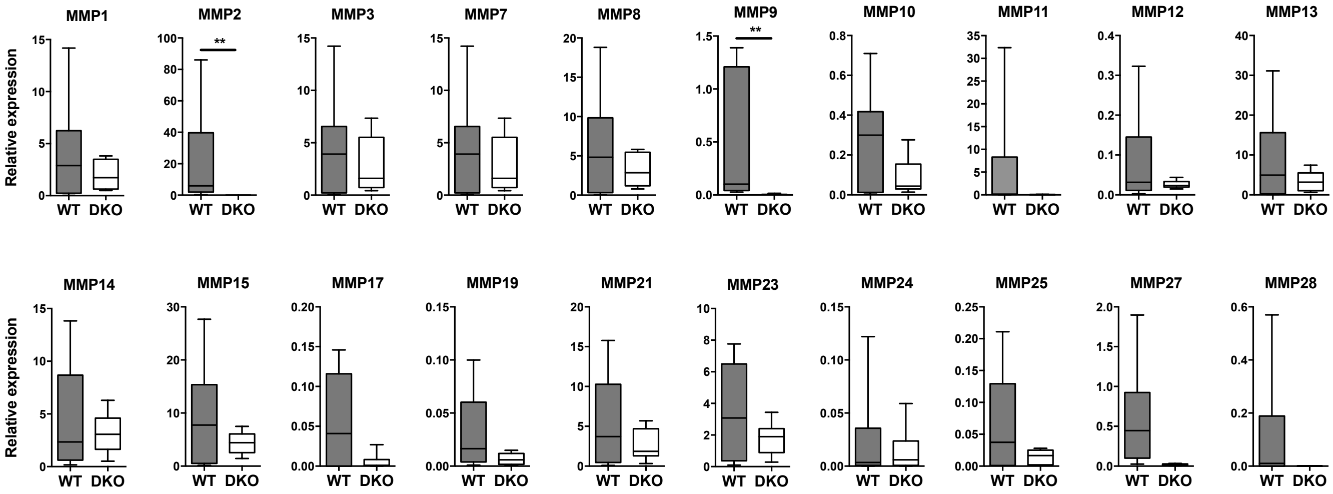

S2 Fig. MMP-2 and -9 deletion was confirmed by RT-PCR. Semi-quantitative RT-PCR showing the gene expression of a panel of MMPs. MMP-2 and MMP-9 are the only MMPs with significant differences between the WT and DKO tumors. N = 6 tumors from PyVmT;WT or PyVmT;DKO mice. Data are box and whisker plots with min and max, ** $p<0.01$, Mann-Whitney test.
