# Supplementary material for: Impact of MMP-2 and MMP-9 enzyme activity on wound healing, tumor growth and RACPP cleavage
Source: PLoS One. 2018 Sep 24;13(9):e0198464. doi: 10.1371/journal.pone.0198464 (PMC6152858; doi:10.1371/journal.pone.0198464)
Supplement: S3 Fig — A. Immunohistochemistry for neutrophil staining (NIMP-R14 antibody) of tumor sections with no primary antibody (control) as well as in WT-T in WT-M and DKO-M. B. The brown staining neutrophils from immunostained tumor sections were counted and represented per mm2 of tumor area (4–6 sections/group). Data are means ± SEM analyzed by Student’s t test. (PDF) [file pone.0198464.s006.pdf]

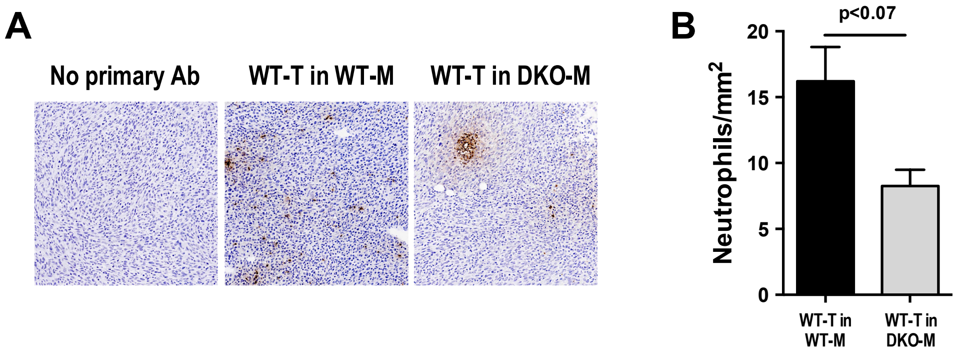

S3 Fig. Neutrophil infiltration in WT and DKO tumors. A. Immunohistochemistry for neutrophil staining (NIMP-R14 antibody) of tumor sections with no primary antibody (control) as well as in WT-T in WT-M and DKO-M. B. The brown staining neutrophils from immunostained tumor sections were counted and represented per mm^2 of tumor area. N = 4-6 sections/group. Data are means ± SEM analyzed by Student's t test.
